# Dental Pupilage

**Published:** 1864-10

**Authors:** A. Lawrence

**Affiliations:** Lowell, Mass.


### DENTAL PUPILAGE.

BY A. LAWRENCE, LOWELL, MASS.

"And how achieved you these endowments, which
You make more rich to owe?"

Every dentist, who has the permanent respectability of his profession at heart, must be interested in the subject of Dental Pupilage.

To usefully and profitably pursue any calling, which anybody ought to engage in, requires previous education—schooling—learning.

If I am not mistaken, a statute law, or at any rate a custom, of England requires a seven years' apprenticeship to any trade, no matter what, and I am not aware that much cause for complaint has grown out of the practical working of such law or custom. On the contrary I hazard the opinion that all parties are benefited *there*, and that the adoption of a similar law or custom in this country would not only tend to give us more really skillful artisans, but do much to moderate that tendency to itinerancy in professions and habitation, which to some extent characterises our people. It is the facility with which any business may be abandoned and another taken up, that leads to so much ignorance and inefficiency in our profession as well as in others. In the absence of any restraint, even by the forms of custom, he who was "Adam Strong, Boot and Shoe Maker," yesterday, is "Dr. A. Strong, Surgeon Dentist," to-day, and it would be difficult to determine whether he or the public are most surprised at the metamorphosis as they read the flaming announcement thereof in the "Village Clarion." Yet Dr. Strong is a very ingenious man, "took it up in his own head," and is entitled to respect and support, particularly so long as he behaves himself, hires a seat in the broad aisle and goes to "our meetin."

I mean no disrespect to the Christian faith in thus making Dr. Strong a church-goer, even if, as is too frequently the case, he does so merely as an advertising medium, but simply to show how little the public in general understand or care about the qualifications of the man to whom they entrust the care of those useful organs, the teeth.

Now is it probable that the dentist graduating as Dr. Strong has, or as many others have, except that they have "*studied*" with Dr. Strong three weeks or as many months, can have possessed him or themselves of that store of theoretical or practical knowledge, in detail, so essential to a useful practice?

I desire to institute no comparisons nor cast reflections upon any member of the profession, who has any business in it, but only to enquire whether we are fully satisfied with the idea that dentistry may be mastered in the short time and with the limited qualifications those wishing to enter our ranks think necessary for the purpose?

The fact is, that those designing to become dentists wish to do so with as little outlay of time or money as possible, and although they declare for the most thorough course are almost uniformly surprised to learn what is required of them as preliminary qualifications, term of pupilage, &c., unless, indeed, they have applied to Dr. Strong. Then the case is different—no familiarity with books—no Anatomy, Chemistry, Physiology, &c.—no wasting time and money in those humbug Dental Colleges, but most frequently consisting in the sale and purchase of certain mysteries and "showing," it being fully understood by the high contracting parties that the sooner the stipulations are fulfilled the better for all concerned.

Now, seriously, we have had enough of such pupilage, and Tom, Dick and Harry turning dentist in a night, or next thing to it.

Enough of this half-way teaching as well as of learning, and those of us now upon the stage, in view of the present improved state of dental science, of which we are justly proud, owe it to the public—the great future—to our pupils as well as ourselves, to inaugurate some much needed reforms in the matter under consideration.

I may properly introduce here as illustrative in part of my own views an article from the By-Laws of the Merrimack Valley Dental Association, organized Oct. 29, 1863:

"Article VI. No member of this association shall take a student for a less term than two years, unless he shall have studied dentistry with some other practitioner, so as to make his term of pupilage equal to two years, or has attended one full course of lectures at some Medical or Dental College."

The American Dental Association at a meeting recently held at Niagara Falls, and the American Dental Convention in session at Detroit a few days after, both passed some resolutions to the same effect, only, I am happy to add, a little stronger.

Now then if we can not make a statute law applicable to the case let us at least establish a custom, that all henceforward may be governed thereby, and thus do something toward further elevating our common fraternity.

